# Evidence for effective interventions to reduce mental health-related stigma and discrimination in the medium and long term: systematic review

**DOI:** 10.1192/bjp.bp.114.151944

**Published:** 2015-11

**Authors:** N. Mehta, S. Clement, E. Marcus, A.-C. Stona, N. Bezborodovs, S. Evans-Lacko, J. Palacios, M. Docherty, E. Barley, D. Rose, M. Koschorke, R. Shidhaye, C. Henderson, G. Thornicroft

**Affiliations:** **Nisha Mehta**, MBBS, **Sarah Clement**, PhD, **Elena Marcus**, MSc, **Anne-Claire Stona**, BSc, **Nikita Bezborodovs**, MD, **Sara Evans-Lacko**, PhD, **Jorge Palacios**, PhD, **Mary Docherty**, MRCPsych, **Elizabeth Barley**, PhD, **Diana Rose**, PhD, **Mirja Koschorke**, PhD, Health Service and Population Research Department, King's College London, Institute of Psychiatry, Psychology and Neuroscience, London, UK; **Rahul Shidhaye**, PhD, Centre for Mental Health, Public Health Foundation of India, Delhi, India; **Claire Henderson**, PhD, **Graham Thornicroft**, PhD, Health Service and Population Research Department, King's College London, Institute of Psychiatry, Psychology and Neuroscience, London, UK

## Abstract

**Background**

Most research on interventions to counter stigma and discrimination has focused on short-term outcomes and has been conducted in high-income settings.

**Aims**

To synthesise what is known globally about effective interventions to reduce mental illness-based stigma and discrimination, in relation first to effectiveness in the medium and long term (minimum 4 weeks), and second to interventions in low- and middle-income countries (LMICs).

**Method**

We searched six databases from 1980 to 2013 and conducted a multi-language Google search for quantitative studies addressing the research questions. Effect sizes were calculated from eligible studies where possible, and narrative syntheses conducted. Subgroup analysis compared interventions with and without social contact.

**Results**

Eighty studies (*n* = 422 653) were included in the review. For studies with medium or long-term follow-up (72, of which 21 had calculable effect sizes) median standardised mean differences were 0.54 for knowledge and −0.26 for stigmatising attitudes. Those containing social contact (direct or indirect) were not more effective than those without. The 11 LMIC studies were all from middle-income countries. Effect sizes were rarely calculable for behavioural outcomes or in LMIC studies.

**Conclusions**

There is modest evidence for the effectiveness of anti-stigma interventions beyond 4 weeks follow-up in terms of increasing knowledge and reducing stigmatising attitudes. Evidence does not support the view that social contact is the more effective type of intervention for improving attitudes in the medium to long term. Methodologically strong research is needed on which to base decisions on investment in stigma-reducing interventions.

Since Goffman's seminal work on stigma,^1^ research in this field has steadily grown,^2^ although most work consists of surveys among the general public about attitudes towards people with mental illness,^3–6^ and much less is known about effective interventions to reduce stigma,^6^ or about stigma in low- and middle-income countries (LMICs).^7–10^ To better understand the evidence base on interventions to reduce mental illness-related stigma and discrimination, we identified eight existing systematic reviews on this topic.^11–18^ The reviews varied widely in their methods and foci. There was considerable methodological and clinical heterogeneity in the included studies, and consequently meta-analysis was only undertaken in one review,^11^ and for small subgroups in two others.^12,13^ Four reviews presented data or commented on the overall pattern of effect sizes,^11–14^ and in each of these the interventions had small to moderate effects, using Cohen's interpretation.^19^ There was clearest consensus that the interventions containing social contact and first-person narratives were more effective than others.^11,13,15,16^ Two of the reviews explored moderators of effects to understand which types of contact work best,^11,13^ but there is a need for more research in this area. Two reviews indicated that some interventions have the potential to worsen stigma.^13,17^ Most of the reviews were critical of the methodological quality of the included studies,^12–15,18^ commenting in particular on the need for more randomised controlled trials (RCTs) and robust methods generally; the use of unvalidated measures; and the relative lack of follow-up beyond the immediate post-intervention period. Other study limitations noted were the use of convenience samples,^13,15,17^ small sample sizes,^14^ or inappropriate outcome measures.^14,15^ Some reviews highlighted the poor quality of the interventions, which were sometimes delivered without training, manualisation or fidelity checks,^11^ and interventions often lacked a theoretical underpinning and developmental research.^13,14^ In all except one review, which was restricted to studies in Iran,^12^ interventions taking place in LMICs were a small minority or did not feature. From this scoping of existing systematic reviews we concluded that there was a need for a further systematic review to synthesise the evidence on two key issues: effectiveness in the longer term and in LMIC contexts. Consequently this systematic review aimed to assess the effectiveness of interventions (of any type with any target population), compared with inactive or baseline comparators, in reducing mental health-related stigma (knowledge, attitudes and behaviour) using any quantitative study design, addressing specifically the evidence for medium- and long-term effectiveness (research question 1) and the effectiveness of interventions in LMICs (research question 2).

## Method

Studies were included if they described any type of intervention with a stated aim of changing mental health-related stigma or with an implied aim of changing stigma as indicated by the inclusion of at least one of the following core stigma-related outcomes: stigma (any), prejudice (attitudes and related outcomes), discrimination, internalised/self-stigma or public mental health awareness/literacy. Intervention studies were included if they related to functional mental illnesses; interventions solely about, or delivered to, populations with dementia, substance misuse, intellectal disabilities or developmental disorders were excluded from this review. We included all quantitative study designs, including RCTs, controlled and uncontrolled pre–post studies, crossover studies, cohort studies and longitudinal panel studies. Studies with more than one intervention group were included. To be eligible, studies needed to report a comparison with a control group (including treatment as usual, best available current treatment or an active control, to control for non-specific effects of the intervention) or a baseline comparator. Studies needed to include at least one stigma outcome which we categorised as related to knowledge, attitudes (prejudice, self- stigma, self-esteem) or behaviour (discrimination, stigma-coping). To be eligible studies also had to address one of our two research questions: to have at least one follow-up point at least 4 weeks after the intervention was completed (to reflect the importance of medium- and longer-term outcomes relevant to stigma, as this is often described by people with mental illness as a long-term challenge); or for the intervention to be carried out in an LMIC setting. Eligibility criteria are shown in the Appendix.

### Information sources and search strategy

**Fig. 1:**
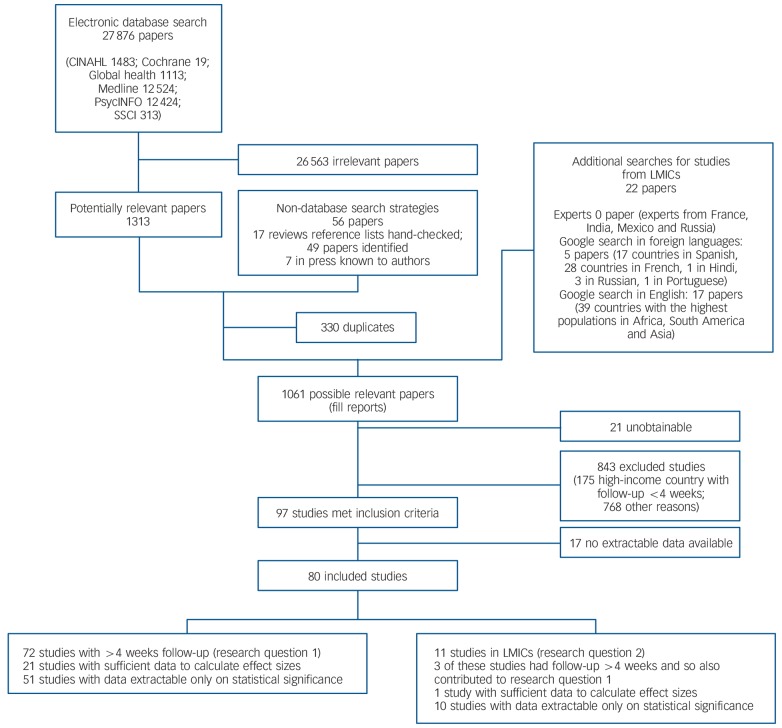
Selection of papers and sources included in the review. LMIC, low- and middle-income country; SSCI, Social Science Citation Index.

We identified studies by searching electronic databases, hand-checking reference lists of reviews and consulting with experts in the working group with knowledge of papers in press. We searched the following databases between 25 January 2013 and 8 February 2013: Medline, PsycINFO, the Cochrane Library, the Cumulative Index to Nursing and Allied Health Literature (CINAHL), the Social Science Citation Index (SSCI) and Global Health. In addition we conducted a Google advanced search focusing on LMICs (see Fig. 1 for details). The Google search was warranted in the light of the limited amount of stigma research in LMICs, but was precluded for our first research question as research from high-income countries is more likely to be found through a standard systematic review search. A search strategy was developed by consensus among authors (N.M., S.C., E.B. and M.D.) using both MeSH and text word searching. We searched using the format ‘Stigma’ OR ‘Discrimination’ OR synonyms AND ‘mental health’ OR ‘mental disorders’ OR synonyms AND ‘Intervention Studies’ OR synonyms. The full Medline search strategy is shown in online Table DS1. The search was restricted to results between 1980 and 2013 and studies on human beings, but was not limited by language. The decision to start the search at 1980 was a pragmatic one based on our examination of the existing reviews which revealed that the vast majority of stigma intervention research commenced after 1980. Relevant non-English language papers were read by fluent native language speakers in French and Spanish according to the linguistic skills available to members of the review team. Potentially relevant papers in many important languages, including Chinese, were therefore excluded from the review. Systematic and non-systematic reviews were identified during the search and the reference lists of these studies were hand-checked.

### Study selection and data collection

All identified titles and abstracts were screened by two researchers. Because of the large number of search hits, two researchers screened 5% of abstracts together. As good agreement (<95%) was achieved, the remainder were divided between the two researchers and study selection conducted by one researcher for each half. Where the researcher was unclear as to whether a paper should be included, the paper was discussed in consensus meetings. Two review authors extracted data from included studies for all parts of the systematic review, with queries resolved by discussion and consensus.

### Statistical analysis

Outcomes for the studies included were reported using both scales and individual items, although for the effect size calculations were restricted to scale data for knowledge and attitudes. We classified all reported stigma outcomes into the categories of ‘knowledge’, ‘attitudes’ or ‘behaviour’. Differences between intervention group and control group at follow-up were our main focus for the quantitative review. Effect sizes, standardised mean differences (SMDs) and 95% confidence intervals were calculated for studies where there were sufficient data to calculate this using the Campbell Collaboration effect size calculator.^20^ We had planned to calculate odds ratios for dichotomous outcomes but found no study for which this was calculable. Negative SMDs indicate a reduction in stigma (benefit), i.e. an improvement in knowledge outcomes or a reduction in either negative attitudes or discriminatory behaviour in the intervention group. Where more than one outcome was reported within a category, the median effect size was presented.^21^ In the online tables we present data on the number of outcomes with statistically significant changes in outcome and the direction of effect to complement the effect size data of outcomes.^21^ These also provide some information about all included studies and at least some information on effectiveness for studies that reported insufficient data to calculate effect sizes. Owing to the considerable heterogeneity of the interventions, measures and participants in the included studies, it was not possible to conduct meta-analyses or to use conventional analytical methods to control for heterogeneity. As some studies had more than one intervention, this analysis was carried out at the intervention level with the number of participants in the control group split between the interventions, to control for unit of analysis error.^22^

We conducted two subgroup analyses on type of intervention by calculating, presenting and comparing median effect sizes attitude outcomes for each subgroup. The first analysis compared direct, indirect or no social contact, and the second compared target groups. We undertook similar sensitivity analyses to explore the possible effects of study design and risk of bias. First, we compared RCT evidence with non-RCT evidence, and second, within RCTs we compared the third of studies with the least risk of bias (see below) with the remainder.

### Assessment of study quality and risk of bias

A quality assessment and profile of risk of bias within studies were carried out individually for all included studies. Level of RCT evidence was rated by two authors using the Cochrane risk of bias tool.^23^ The third of RCTs with the lowest risk of bias are identified with an asterisk in the data extraction tables. To assess bias in non-randomised studies two researchers conducted quality appraisals using risk of bias criteria for non-randomised studies,^23^ suitable to the wide range of study designs included. When a decision about the risk of bias could not be made, it was resolved through discussion with a third author. In addition, for each study we indicated whether at least one outcome measure was validated, whether it was previously published, developed by the author or if items were used. Scales were marked as having evidence of psychometric adequacy providing they met one or more of the following criteria: the authors reported a Cronbach's α of 0.7 or greater, the authors referenced the measure as being reliable or valid, or there was some evidence of validity or reliability as judged by the review team.

## Results

A total of 80 quantitative studies (422 653 participants) were identified for inclusion in the review, 72 addressing research question 1 (long-term effectiveness) and 11 addressing research question 2 (setting), of which 3 studies addressed both questions (Fig. 1). The database search provided 27 876 citations. After a review of the abstracts 26 563 papers were excluded as they were clearly irrelevant or did not meet the inclusion criteria. The reference lists of 17 reviews were hand-checked and 49 further papers identified. Seven papers in press known to the authors were included. After removal of 330 duplicates the full text of the remaining 1061 potentially relevant papers was sought. Of these, 21 papers were unobtainable and 843 papers did not meet the inclusion criteria. Of the remaining papers 17 did not contain enough relevant data to extract. A full reference list is given in online Table DS2 and study characteristics are listed in online Tables DS3–5. Online Tables DS6 and DS7 give risk of bias and quality ratings for RCTs and other studies respectively.

### Medium- and long-term follow-up

#### Study characteristics

Most of the studies addressing medium- or long-term outcomes took place in high-income countries (93%), were aimed at school or university students (37%) and used interventions comprising mental health education and literacy or mental health information (43%). About a quarter (28%) of the studies included were RCTs, 52% consisted of pre–post studies with or without a control group and 21% were longitudinal panel or cohort studies. Most studies (69%) had a final follow-up assessment 1–6 months after the intervention had ended, whereas 21% had a longer follow-up (1–10 years post-intervention). Tables DS3 and DS4 show details of study characteristics.

#### Evidence

There were 72 quantitative studies with at least 4 weeks of follow-up, which included 81 interventions with 42 653 participants. It was possible to calculate effect sizes and confidence intervals for 21 of these studies (23 interventions). These studies and their effect sizes are shown in Table 1. Findings based on statistical significance for all included studies are shown in Tables DS3 (RCT, controlled and uncontrolled pre–post studies) and DS4 (longitudinal panel study or cohort design). For knowledge outcomes the median effect size was 0.54 indicating a medium effect in increasing knowledge.^19^ For attitude outcomes SMDs ranged from 0.05 to −1.22 with a median effect size of −0.26, indicating a small reduction in stigmatising attitudes. For behavioural outcomes SMDs were calculated in one intervention which showed a small (SMD = 0.22) effect in reducing stigmatising behaviour. Inspection of the pattern of significance findings for scales for all the included studies with medium- or long-term follow-up indicated that there were similar numbers of significant and non-significant findings indicating an increase in knowledge (26 *v*. 22). Similar numbers were also found for attitude scales (63 non-significant findings *v*. 52 significant in the direction of stigma reduction). Five scales had significant findings indicating an increase in stigma. For behavioural outcomes measured with scales, non-significant findings out-numbered significant ones indicating a reduction in discriminatory behaviour (12 *v*. 2) and this was also the case for behavioural outcomes measured at the item level (38 *v*. 19).

**Table 1:** Evidence for medium- and long-term effectiveness of interventions to reduce mental health-related stigma

					Evidence for effectiveness SMD (95% CI)^e^		
Study^a^	Design^b^	*n*^c^	Intervention	Time to follow-up^d^	Knowledge	Attitudes	Behaviour
Targeted at the armed forces							
Seal et al (2012)^45^	RCT	73	Motivational interviewing	8 weeks		0.04 (−0.07 to 0.86)	
Gould *et al* (2007)^46^	Controlled	124	Training programme to provide support, education and modify attitudes about PTSD	1 month		0.42(r) (0.00 to 0.85)	
Targeted at school students							
Campbell *et al* (2011)^47^	RCT	92	Mental health workshop including education and direct contact	10 weeks		0.05 (−0.39 to 0.49)	
Pinto-Foltz *et al* (2011)^48^	RCT	156	Direct contact with service users who were in sustained recovery from mental illness	8 weeks	0.29 (-0.05 to 0.63)	−0.17 (−0.50 to 0.17)	
Esters *et al* (1998)^49^	Controlled	40	Mental health education about stigma and help-seeking	12 weeks		**−0.45 (−1.08 to −0.18)**	
O'Kearney *et al* (2006)^50^	Controlled	59	Internet programme aiming to help people identify, overcome and cope with depression	16 weeks		−0.25 (−0.83 to 0.34)	
O'Kearney *et al* (2009)^51^	Controlled	157	Internet programme aiming to help people identify, overcome and cope with depression	20 weeks	−0.14 (−0.45 to 0.18)	**−0.17 (−0.49 to 0.15)**	
Ventieri *et al* (2011)^52^	Controlled	195	Mental health education, with role play and activities	4 months	**0.51 (0.21 to 0.80)**	**−0.33 (−0.62 to −0.03)**	
Targeted at university students							
Gonzales *et al* (2002)^53^	RCT	167	Mental health education about stigma	4 weeks		−0.07 (−0.52 to 0.38)	
Sharp *et al* (2006)^54^	RCT	123	Mental health education	1 month		−0.09 (−0.47 to 0.29)	
Faigin & Stein (2008)^55^	Controlled	204	A play by actors with history of severe mental illness addressing their experiences and stigma	1 month		−0.13 (−0.47 to 0.20)	
Faigin & Stein (2008)^55^ (2nd arm)	Controlled	222	A video-recorded version of the play described above	1 month		−0.37 (−0.69 to −0.05)	
O'Reilly *et al* (2011)^56^	Controlled	272	Mental health first aid training for pharmacy students	6 weeks		−0.61 (−0.92 to −0.31)	
Targeted at healthcare professionals							
Blair Irvine *et al* (2012)^57^	RCT	172	Internet courses with behavioural skills and knowledge training for long-term care staff	1 month	**0.56 (0.25 to 0.86)**	**−0.17 (−0.47 to 0.13)**	
Patterson *et al* (2007)^58^	Controlled^f^	91	Educational intervention about self-harm behaviour for nurses	18 months		−1.22 (−1.86 to −0.58)	
Treloar (2009)^59^	Controlled^f^	90	Educational programme about self-harm using psychoanalytic aetiology framework	6 months		−0.35 (−1.06 to 0.37)	
Treloar (2009)^59^ (2nd arm)	Controlled^f^	91	Educational programme about self-harm using CBT aetiology framework	6 months		−0.47 (−1.23 to 0.29)	
Targeted at the general public							
Jorm *et al* (2004)^60^	RCT*	753	Mental health first aid course	4 months	**11.77 (5.98 to 17.56)**	**−0.26 (−0.49 to −0.03)**	0.22(r) (−0.18 to 0.63)
Targeted at people with mental health problems							
Fung *et al* (2011)^61^	RCT*	66	Self-stigma reduction programme	6 months		0.34 (−0.82 to 0.15)	
Gumley *et al* (2006)^62^	RCT	144	CBT targeting negative beliefs about self and illness	12 months		−0.12 (−0.45 to 0.21)	
Targeted at other groups							
Gulliver *et al* (2012)^24^	RCT*	59	Mental health literacy and destigmatisation intervention for elite athletes	3 months	0.76 (−0.17 to 1.68)	0.50(r) (0.41 to 1.41)	
Kitchener & Jorm (2004)^27^	RCT*	301	Mental health first aid course for employees	5 months	0.07 (−0.16 to 0.30)	-0.17 (−0.40 to 0.05)	
Jorm *et al* (2010)^63^	RCT*	327	Youth mental health first aid course for teachers	6 months	0.67 (0.18 to 0.65)		

CBT, cognitive–behavioural therapy; PTSD, post–traumatic stress disorder; RCT, randomised controlled trial; SMD, standardised mean difference.

a. Studies with sufficient data to calculate effect sizes.

b. Designs include RCTs in the top tercile for quality, i.e. highest numbers of Cochrane risk of bias items rated as low (RCT*); RCTs in the lower two terciles for quality (RCT (see online Table DS5 for details); pre–post studies with a control group (Controlled).

c. Number of participants in the intervention and control groups.

d. Time to final follow-up results.

e. An SMD <0 indicates a reduction in knowledge, stigmatising attitudes or stigmatising behaviours unless the data are such that this can only be calculated to show the reverse effect, in which case this is marked (r). Bold type indicates confidence intervals that do not cross zero.

f. Hedges' *g* used by study authors instead of Cohen's d owing to small sample sizes.

Our subgroup analysis of type of intervention found that interventions containing direct social contact had a smaller median effect size for stigmatising attitudes (−0.17) than those with indirect social contact (−0.32) or no social contact (−0.33). There were enough interventions with effect sizes to make comparisons of median effect sizes by three types of target group, and we found that interventions targeted at health professionals had a somewhat higher median effect size (−0.41) than those targeting school pupils (−0.21) or university students (−0.13).

#### Risk of bias

Across all RCTs there was a low risk of bias for 50% of the criteria and an unclear or high risk of bias in the other 50%. Only five trials met 70% or more of the criteria. Nine trials met between 40% and 60% of the criteria and five only met 15–30%. In light of the nature of anti-stigma interventions it was not possible to mask participants and personnel to allocation, with the exception of one trial which was internet-based and thus easier to conceal.^24^ Of the 19 trials, 17 used at least one validated scale to measure outcomes, whereas 2 used non-validated scales that had been used in previously published papers. There were 53 non-randomised studies, 30 of which did not have a control group. Among studies with a control group, 6 were deemed to have a low risk of selection bias with regard to the comparability between the intervention and control groups. In 26 studies there was a high risk of attrition bias, where more than 20% of the sample were lost to follow-up and no intention to treat analysis was carried out. Possible confounders were considered and controlled for in only 28% of studies. As with the RCTs, masking of participants and personnel was not possible owing to the type of intervention. Among non-randomised studies, 24 had at least one validated outcome measure, 2 had at least one that was previously published, 4 had one that was specifically developed for the study with no psychometric testing reported, and 23 used items only. Details of risk of bias in individual studies are given in Tables DS5 and DS6. The median effect size for RCTs was lower than for non-randomised controlled studies (−0.17 *v*. −0.37). Within RCTs the third with the least risk of bias had a higher effect size (−0.30) than the remainder (−0.09).

### Evidence from LMICs

There were 11 studies (1967 participants) from LMIC settings, 8 with less than a 4-week follow-up and 3 with longer follow-up. Study characteristics and statistical significance findings for these are shown in Table DS5. Eight of these were from upper middle-income countries and three were from lower middle-income countries. There was no study meeting our criteria from a low-income country. Six studies were aimed at school and university students, two at caregivers of people with schizophrenia, and three at healthcare professionals. Three studies used an RCT design, one of which was a cluster randomised trial analysed within groups, two were controlled studies and six were uncontrolled pre–post studies. Within the 11 studies included there were 16 intervention arms, with 5 measuring knowledge outcomes and 14 measuring attitude outcomes. None of the studies had behavioural outcomes. Sufficient data to calculate an effect size were reported in only one of the studies;^25^ in this study – a psychoeducation programme for caregivers of patients with schizophrenia in Chile – the SMD for stigmatising attitudes was −2.11 (95% CI −2.87 to −1.34), indicating a large effect. Inspection of the statistical significance of the knowledge scale findings for all studies revealed that both studies with such outcomes found no evidence of change; however, there were findings indicating a significant reduction in stigmatising attitudes for 11 of the 12 attitude scale outcomes assessed in these studies (Table DS5).

These results should be interpreted with caution. In seven of the studies, follow-up assessments were undertaken immediately after the intervention (in one study this was done 1 week after the intervention had ended). There were also issues regarding bias: owing to a lack of information in the papers it was generally difficult to gauge the extent of risk of bias. For the three RCTs, in 52% of criteria the risk of bias was unclear. Where information was provided, a high risk of bias was found in 19% of criteria across the RCTs, whereas in 29% of criteria the risk was low. This was most common for the incomplete outcome data and selective outcome reporting criteria. For the non-randomised studies, risk of bias varied across criteria, with 33% classified as high and 33% as low, and for 33% the degree of risk was unclear.

### Behavioural outcomes

Among the 15 studies that did report behavioural outcomes, 7 assessed contact with someone with a mental health problem, 4 measured perceived discrimination and coping strategies in participants who had a mental health problem, 2 measured changes in school and workplace policies regarding mental health,^26,27^ 2 measured experienced discrimination reported by people with mental health problems,^28,29^ and only 1 measured actual discriminatory behaviour by participants in the general population.^30^

## Discussion

Our synthesis of 72 studies with follow-up beyond 4 weeks revealed that, at this follow-up, interventions aimed at reducing mental health-related stigma typically had a medium-sized effect on knowledge outcomes and a small effect on attitudinal outcomes, although for both types of outcome statistically non-significant findings were as common as significant ones. There were insufficient data on behavioural outcomes to draw any conclusions on the medium- or long-term effectiveness of interventions to reduce discrimination. This is the first systematic review to synthesise evidence on medium- and long-term effectiveness, which is striking given that stigma is often experienced by people with mental illness as a long-term difficulty. Although a number of systematic reviews indicated that social contact interventions were particularly effective,^11,13,15,16^ the majority of studies in these reviews had only short-term follow-up. Our review, restricted to studies with medium- and longer-term outcomes, did not support the superiority of social contact interventions as we had expected. As it is vital that stigma reduction is sustained in the longer term, the effectiveness of such social contact interventions clearly warrants further research.

Study quality was variable, and indeed study design and quality did appear to affect median effect sizes, although these subgroup and sensitivity analysis findings should be interpreted with caution owing to the heterogeneity of the studies. Overall, where we did identify positive changes from the interventions, the magnitude of the effects was generally rather modest. It is also clear that there is therefore a lack of research on actual discriminatory behaviour within the stigma research field.

For our second research question regarding LMICs, we found comparatively few studies from middle-income countries and none from low-income countries. A large effect size was found for the one LMIC study for which there were sufficient data to calculate the effect size and the majority of attitude scale outcomes indicated significant improvements in attitudes, although such findings must be treated with considerable caution. There is a clear need for more stigma reduction studies, particularly from low-income countries.

Our results regarding service user social contact are consistent with those of Griffiths *et al*,^31^ who recently published a meta-analysis of RCTs of interventions intended to reduce stigma. Analysing data from 26 trials they found that interventions targeting personal stigma or social distance yielded small but significant reductions in stigma across all mental disorders. Further, they reported that educational interventions were effective in reducing personal stigma, as were interventions incorporating service user contact. This study also considered internet use and self-stigma and found that internet programmes were at least as effective in reducing personal stigma as face-to-face delivery (see also Clement *et al*).^32^ They found no evidence that stigma interventions were effective in reducing self-stigma. In our review, although social contact appears to be the most strongly evidence-based type of intervention to reduce stigma when measured by immediate post-intervention outcomes, there is not at present evidence to show that such immediate benefits persist in the longer term.

### Limitations of the study

This review has a number of limitations. In conducting a comprehensive overview of all relevant literature we have identified considerable heterogeneity among participant groups, interventions and outcomes. For example, we identified 55 different scales used for the 136 outcomes measured. Study quality also varied considerably. We were able to include studies in some non-English languages, but it is possible that we missed important projects published in other languages, for example potentially important studies not available at all in English, or studies for which only abstracts were available in English, and which we were not able to assess fully (see, for example, Shi *et al*).^33^ The temporal limitation of the search start date being 1980 will have resulted in the review missing studies before that date. We also need to acknowledge the possibility of publication bias, for example that intervention studies showing no difference might be published less often than those that do identify a clear benefit. Further, the risk of bias results given above, with half of all studies having a high or unknown risk of bias, mean that considerable caution needs to be exercised in interpreting these findings. It is also notable that relatively few of the interventions assessed following published, manualised procedures or including any rating of treatment fidelity. It should also be appreciated that although a narrative review may be able to disaggregate the nature of the interventions, and the specific target groups, into a greater number of specific subtypes, the numbers of studies in each of these categories would be small, and that this would give a greater descriptive richness at the expense of the wider generalisability of the findings. The systematic review method used here does not allow this narrower focus.

### Challenges in the measurement of stigma

The assessment and validation of instruments to measure stigma and discrimination against people with mental illness has been under way since the 1960s. Although early measures such as the Opinions About Mental Illness and the Community Attitudes to Mental Illness scales are still used in some studies,^34,35^ there have been many developments in the breadth and quantity of measures to assess stigma in recent years. These include a trend to incorporate multiple outcomes or domains, for example knowledge and behaviour as well as attitudes; techniques to control for social desirability bias such as implicit measures; research on coping or ‘stigma resilience’; and assessments among multiply stigmatised groups, such as people from ethnic minorities with mental illness. Despite these developments there are still substantial gaps in what can be assessed using available measures, including a lack of behavioural and structural indicators. We have seen in this review that behaviour is under-represented in stigma intervention outcomes, for example changes in behaviour of others rated by patients or service users, or directly observed discrimination-related outcomes. There is a further gap in terms of important subgroups. For example, Link *et al* noted that children and adolescents were represented in only 3.7% of stigma studies.^36^ More specific and tailored measures might facilitate inclusion of specific subpopulations in stigma research, such as those already affected by discrimination on the grounds of (for example) ethnicity. Additionally, studies that include measures validated in LMICs are rare, and only a few include any intervention component developed specifically in such countries. Future efforts should therefore address these gaps, because measurement and evaluation are critical to understanding the underlying mechanisms and effectiveness of anti-stigma interventions. A further challenge is to stop the use of unvalidated measures and item level analyses, while retaining enough flexibility to promote conceptual, contextual and theoretical relevance.

### Gaps in the evidence base

This review has highlighted clear gaps in the field of anti-stigma interventions and research methods and a need for the harmonisation of outcomes in this field of research. These include the paucity of evidence on discrimination outcomes, or on reducing negative behaviours or increasing positive behaviours towards people with mental illness,^37^ and the lack of studies of specific target groups such as employers or family members, despite service users commonly reporting experiencing discrimination from both of these groups.^38^ There is an important need to assess whether benefits identified in the short term are maintained in the longer term, and if any booster interventions are needed to achieve sustainability. This review has also shown a relatively narrow focus of work to date: either on the general population (in attitude surveys) or on students within settings accessible to researchers (e.g. universities and colleges).^16^ From a global health viewpoint there is a distinct lack of interventional research in LMICs, despite emerging evidence of the scale and severity of the challenges posed by stigma and discrimination, and despite the fact that 85% of the world's population live in such countries.^39,40^ Finally, there is a need for more studies using high-quality research designs. Only a third of studies included in this paper used an RCT or other robust study design, and many of these had a high risk of bias.

### Future research

Knowledge in this field is generally from small studies of poor methodological quality, using inconsistent outcomes scales, and in particular few strong RCTs or interrupted time series studies have been carried out to test interventions intended to reduce stigma and discrimination. Our summary of previous systematic reviews does tend to support the view that social contact is the more effective type of intervention known to reduce stigma, at least in the short term.^41^ We do not yet have even weak consistent evidence to support interventions for target groups identified as priorities by service user groups, such as family members, and only an embryonic evidence base concerning how to address stigma in healthcare staff.^42^ Indeed, this degree of evidential neglect could itself be seen as a manifestation of structural discrimination. Given the magnitude of the challenges posed by stigma and discrimination, it is clear that there needs to be a commensurate concerted effort to fund methodologically strong research to provide robust evidence to support policy decisions on investment and interventions. Such a wider policy framework is now emerging.^43^ The World Health Organization Mental Health Action Plan, ratified by the World Health Assembly in May 2013, states as its vision:

‘A world in which mental health is valued, promoted and protected, mental disorders are prevented and persons affected by these disorders are able to exercise the full range of human rights and to access high quality, culturally-appropriate health and social care in a timely way to promote recovery, in order to attain the highest possible level of health and participate fully in society and at work, free from stigmatization and discrimination.’^44^

Specifically, paragraph 75 of the Action Plan indicates a need to prioritise:

‘Mental health promotion and prevention: provide technical support to countries on the selection, formulation and implementation of evidence-based and cost-effective best practices for promoting mental health, preventing mental disorders, reducing stigmatization and discrimination, and promoting human rights across the lifespan.’^44^

This review indicates that an early necessity is to conduct more high-quality research to allow this policy priority to be firmly evidence-based, especially within LMICs.

## Appendix

### Eligibility criteria for study inclusion

#### Participants

Any, except target populations that solely comprised people with dementia, substance misuse, intellectual disabilities or developmental disorders.

#### Setting

Any.

#### Intervention

Any intervention with a stated aim of changing mental health-related stigma, or with an implied aim of changing stigma as indicated by the inclusion of at least one of the following stigma-related outcomes: stigma (including internalised stigma), prejudice (attitudes and related outcomes), discrimination, or public mental health awareness/mental health literacy. Interventions relating to functional mental illnesses were included, those solely about dementia, substance misuse, learning disabilities or developmental disorders were excluded.

#### Comparison

Inactive or baseline comparator.

#### Outcomes

Outcomes comprising:

- knowledge
- attitudes (prejudice/self-attitudes)
- behaviour (discrimination/stigma-coping)
- follow-up at least 4 weeks after the intervention was completed (research question 1) or any (research question 2).

#### Study design

Any quantitative design.
